# Rapid-reaction kinetics of the butyryl-CoA dehydrogenase component of the electron-bifurcating crotonyl-CoA-dependent NADH:ferredoxin oxidoreductase from *Megasphaera elsdenii*

**DOI:** 10.1016/j.jbc.2023.104853

**Published:** 2023-05-21

**Authors:** Wayne Vigil, Derek Nguyen, Dimitri Niks, Russ Hille

**Affiliations:** Department of Biochemistry, University of California, Riverside, Riverside, California, USA

**Keywords:** butyryl-CoA dehydrogenase, electron transfer, electron-transferring flavoprotein, flavin-based electron bifurcation, rapid-reaction kinetics

## Abstract

We have investigated the equilibrium properties and rapid-reaction kinetics of the isolated butyryl-CoA dehydrogenase (bcd) component of the electron-bifurcating crotonyl-CoA-dependent NADH:ferredoxin oxidoreductase (EtfAB–bcd) from *Megasphaera elsdenii*. We find that a neutral FADH• semiquinone accumulates transiently during both reduction with sodium dithionite and with NADH in the presence of catalytic concentrations of EtfAB. In both cases full reduction of bcd to the hydroquinone is eventually observed, but the accumulation of FADH• indicates that a substantial portion of reduction occurs in sequential one-electron processes rather than a single two-electron event. In rapid-reaction experiments following the reaction of reduced bcd with crotonyl-CoA and oxidized bcd with butyryl-CoA, long-wavelength-absorbing intermediates are observed that are assigned to bcd_red_:crotonyl-CoA and bcd_ox_:butyryl-CoA charge-transfer complexes, demonstrating their kinetic competence in the course of the reaction. In the presence of crotonyl-CoA there is an accumulation of semiquinone that is unequivocally the anionic FAD•^−^ rather than the neutral FADH• seen in the absence of substrate, indicating that binding of substrate/product results in ionization of the bcd semiquinone. In addition to fully characterizing the rapid-reaction kinetics of both the oxidative and reductive half-reactions, our results demonstrate that one-electron processes play an important role in the reduction of bcd in EtfAB–bcd.

Complex flavoenzymes catalyzing electron bifurcation, defined as the splitting of a median-potential pair of reducing equivalents into individual high- and low-potential electrons in a way that is overall thermodynamically favorable, provide the means by which many organisms generate the low-potential reducing equivalents needed for carbon and nitrogen fixation, as well as a variety of other endergonic metabolic processes in a variety of organisms (1, 2). Thermodynamically, flavin-based bifurcation makes use of the capacity of the isoalloxazine ring to undergo both one- and two-electron processes. The flavin ring has three oxidation states: a fully oxidized quinone (FAD [Q]), a one-electron reduced semiquinone (neutral FADH• or anionic FAD•^−^ [SQ], depending on the pK_a_), and a fully reduced hydroquinone (at neutral pH, FADH^−^ [HQ]). The flavin half-potentials in bifurcating systems are highly crossed, with the SQ/HQ half-potential being much higher than the midpoint potential for the two-electron process and the Q/SQ half-potential much lower. As a result, the very low-potential semiquinone oxidation state is highly unstable thermodynamically, a condition considered critical for bifurcation. In the course of bifurcation, the first electron leaving the fully reduced HQ, having been reduced by, *e.g.*, NAD(P)H, has a high reduction potential, with the second, low-potential electron remaining as the unstable FAD•^−^ (3). It is this second, low-potential electron that is used to reduce low-potential acceptors such as ferredoxin or flavodoxin, the high-potential electron ultimately being passed on to high-potential substrates such as crotonyl-CoA, caffeyl-CoA, pyruvate, menaquinone, and ubiquinone (1, 2). A key feature of electron bifurcation is that reduction of the high- and low-potential substrates is tightly coupled, with little or no leakage of the low-potential electron into the high-potential pathway (4, 5). Such leakage is highly favorable thermodynamically, and the basis for the tight coupling observed experimentally must therefore be kinetic rather than thermodynamic in origin.

A subset of bifurcating flavoproteins consists of systems that possess an electron-transferring flavoprotein (ETF) component. ETFs from a variety of prokaryotes and eukaryotes, including humans, have been extensively studied, although most are not involved in bifurcation. These simpler ETFs are αβ dimers, with FAD in the α subunit and AMP in the β subunit; the flavin half-potentials are highly uncrossed with the semiquinone oxidation state, typically the FAD•^−^ anion, highly stabilized. Bifurcating ETFs are also αβ dimers, with both subunits exhibiting significant sequence homology to the nonbifurcating proteins, but possess a second equivalent of FAD in the β subunit in place of the AMP found in nonbifurcating ETFs. It is this second FAD that is the site of electron bifurcation (1, 2). The crotonyl-CoA-dependent NADH:ferredoxin oxidoreductase EtfAB–bcd (for butyryl-CoA dehydrogenase, bcd) from organisms such as *Megasphaera elsdenii*, *Clostridium difficile*, and *Acidaminoccocus fermentans* is an example of an ETF-dependent bifurcating flavoprotein, catalyzing the following reaction:

$${2\;NADH+2Fd}_{ox}+crotonyl-CoA⇌{2\;NAD}^{+}{+2\;Fd}_{red}+butyryl-CoA$$

The bcd component of these systems has a third equivalent of FAD that is the site of crotonyl-CoA reduction, with ferredoxin being reduced directly by the low-potential FAD•^−^ of the bifurcating flavin that is generated in the course of bifurcation. The butyryl-CoA generated from the high-potential pathway is ultimately converted to caproyl-CoA, an intermediate in reverse β-oxidation that occurs in the course of the synthesis of straight-chain fatty acids and other metabolites.

The crystal structure of the EtfAB–bcd complex from *C. difficile* (6), a close homolog for the complex found in *M. elsdenii*, has been determined and provides a structural context in which to understand electron bifurcation. The *C. difficile* EtfAB–bcd is a tetramer of heterotrimeric protomers, with four EtfAB subunits surrounding a (bcd)_4_ core. The quaternary structure of the enzyme is a dimer of dimers, with the bcd subunits of one dimer interacting with one another to a greater extent than with the corresponding bcd subunits of the other pair. The domain containing the electron-transferring FAD (hereafter et FAD) is oriented with its flavin in close proximity to that of its partner bcd subunit, and >30 Å from the bifurcating FAD of the EtfAB. This orientation contrasts with that seen in the crystal structure of EtfAB alone from *A. fermentans* (7), where in the absence of the bcd component the et FAD lies in close proximity to the bifurcating FAD. It is evident that the domain containing the et FAD has substantial mobility within the complex, being able to rotate ∼60 degrees between the bifurcating FAD and the FAD of the bcd subunit (6). This motion is thought to be critical for transfer of reducing equivalents from the bifurcating FAD to the bcd FAD and appears to be made possible by the stabilizing interactions between the bcd subunits of the (EtfAB–bcd)_2_ pairs within the tetramer. The conformational flexibility of the domain possessing the et FAD has also been observed previously in nonbifurcating ETFs (8), and appears to be a common property of all electron-transferring flavoproteins.

Interestingly, both the EtfAB and bcd components of the *M. elsdenii* EtfAB–bcd had been studied independently of one another long before their mutual involvement in electron bifurcation was recognized. The EtfAB component was first purified in 1974 (9) and was immediately recognized as possessing two equivalents of FAD rather than just one as seen in other ETFs known at the time. It was shown to catalyze reduction of butyryl-CoA dehydrogenase, but this was seen to simply be analogous to the well-characterized capacity of simple, single-FAD ETFs to catalyze reduction of acyl-CoA dehydrogenases. Subsequent work established an unusual red shift in the absorption envelope of the electron-transferring FAD and demonstrated that it could be reversibly removed from EtfAB by incubation with KBr (10). Reduction potentials were also determined, yielding values of +81 mV and −136 mV for the Q/SQ and SQ/HQ couples of the electron-transferring FAD and −279 mV for the two-electron Q/HQ midpoint potential for the second (bifurcating) FAD (11).

The bcd of *M. elsdenii* had been somewhat less extensively examined prior to recognition of its role as part of an electron bifurcating system. Early preparations were complicated by variable amounts of a “green” form of the enzyme exhibiting long-wavelength absorption at 710 nm that turned out to be due to a charge-transfer complex between oxidized enzyme and a CoA persulfide (12). Mechanistic work demonstrated that reduction of enzyme by butyryl-CoA involved direct hydride transfer from the β carbon after proton abstraction at the α carbon forming a carbanion intermediate (13, 14). A rate of reoxidation of reduced bcd by crotonyl-CoA of 2.6 s^−1^ was reported (14), but without reference or experimental support. The midpoint potential of the *M. elsdenii* bcd was determined to be −79 mV, some 40 mV more positive than the −13 mV potential for the crotonyl-CoA/butyryl-CoA couple (15). Binding of the substrate analogue acetoacetyl-CoA was found to lower the protein midpoint potential by 100 mV (15), but that binding of butyryl-CoA raised the enzyme midpoint potential instead, by some 60 mV (16). Finally, the crystal structure of the *M. elsdenii* bcd was determined and shown to exhibit significant structural homology to the porcine medium-chain acyl-CoA dehydrogenase, with substrate binding occurring on the *re* face of the enzyme FAD (17). The *si* face of the FAD was found to be quite solvent exposed, accounting for the relatively high rate of reaction of the reduced protein with O_2_ (unusual for a dehydrogenase).

Surprisingly, no comprehensive study of the rapid-reaction kinetics of the isolated bcd has yet been reported. In order to characterize the kinetic behavior of the EtfAB–bcd system, we have characterized the reaction of NADH with EtfAB in previous work (18, 19) and here have initiated a rapid-reaction kinetic study of the bcd component, taking advantage of recombinant expression systems for the separate components. Our results point to a role for one- as well as two-electron processes in the reduction of bcd by EtfAB. In addition, we establish that previously identified charge-transfer complexes between bcd and substate/product are kinetically competent in the reoxidation reaction.

## Results

### Reductive titrations of bcd with sodium dithionite, NADH, and butyryl-CoA

**Figure 1:**
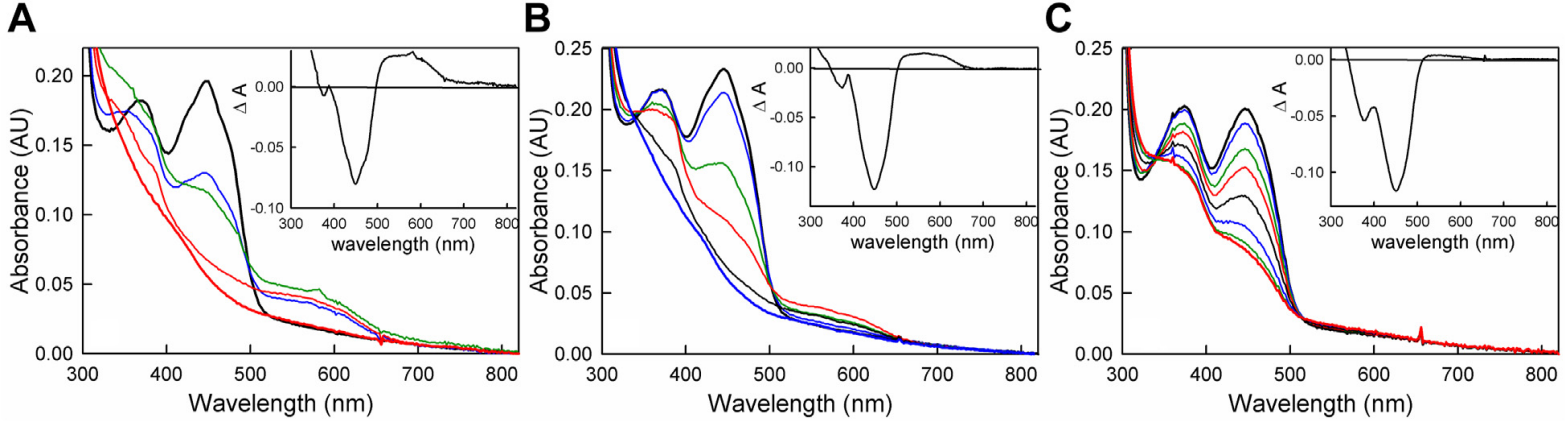
**Reductive dithionite titrations of bcd as a function of pH.***A*, titration at pH 6.0 of 17 μM bcd (*bold black*) in 50 mM MES, 150 mM NaCl, pH 6.0. *Green* and *bold red spectra* represent the points of largest accumulation of FADH• and full reduction, respectively, over the course of the titration. *Inset*, the difference spectrum between the point of greatest FADH• accumulation and that of oxidized enzyme. *B*, titration at pH 7.5 of 19 μM bcd (*bold black*) in 50 mM Tris-HCl, 150 mM NaCl, pH 7.5. *Red* and *bold blue spectra* represent the points of greatest accumulation of FADH• and full reduction, respectively. *Inset*, the difference spectrum of the point of largest FADH• accumulation minus that of oxidized enzyme. *C*, titration at pH 9.0 of 17 μM bcd (*bold black*) in 50 mM TAPS, 150 mM NaCl, pH 9.0. *Inset*, the difference spectrum between the point of greatest FADH• accumulation and that of oxidized enzyme. In all three insets, the negative features at ∼450 nm reflect flavin reduction and the positive absorption in the 500 to 600 nm range reflects FADH• accumulation, this being greatest at pH 6.0 and least at pH 9.0. All experiments were performed at 25 °C.

As reported previously (15, 19), during reductive titrations of bcd with sodium dithionite at pH 7.5 the one-electron reduced FAD neutral semiquinone (FADH•) accumulates transiently with a characteristically broad long wavelength absorbance having a maximum at approximately 571 nm (Fig. 1); this accumulation of FADH• is confirmed by electron paramagnetic resonance (EPR) (see below) and amounts to 16% total accumulation, in good agreement with the value of 19% seen previously (15). We have also undertaken titrations of bcd with sodium dithionite at pH 6.0 and 9.0 and find that, as expected, FADH• accumulates to a significantly greater extent in the course of the reductive titration at pH 6.0 than at pH 7.5, and less so at pH 9.0 (Fig. 1).

**Figure 2:**
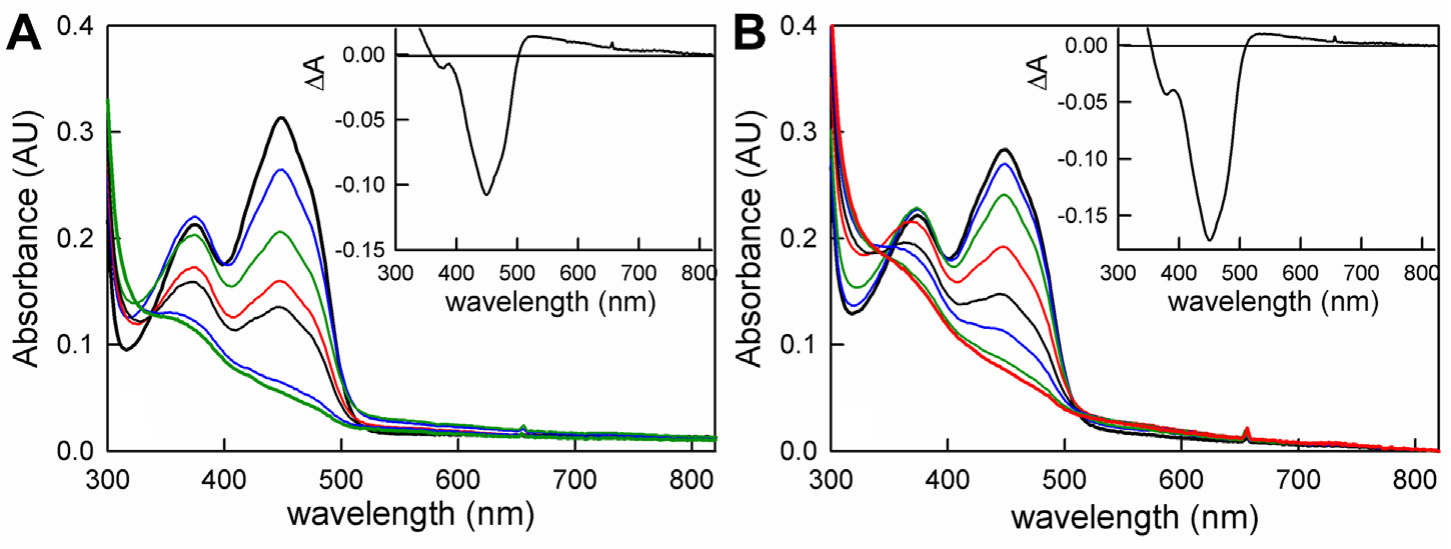
**Reduction of bcd in the presence of catalytic amounts of EtfAB.***A*, reductive titration with sodium dithionite of 27 μM bcd (*bold black*) and 1 μM EtfAB. *Inset*, the difference spectrum between the point of largest accumulation of FADH• and that of oxidized enzyme. *B*, reductive titration with NADH of 25 μM bcd (*bold black*) and 1 μM EtfAB. *Inset*, the difference spectrum between the point of largest accumulation of FADH• and that of oxidized enzyme. The negative features at 450 nm reflect flavin reduction, and the positive features above 500 nm reflect FADH• accumulation. Both titrations were performed in 50 mM Tris-HCl, 150 mM NaCl, pH 7.5, 25 °C.

We next performed reductive titrations of oxidized bcd in the presence of a catalytic amount of EtfAB with both sodium dithionite and NADH at pH 7.5. As seen in Figure 2, we do not observe any major differences between the two titrations, although there is somewhat greater accumulation of FADH• in the dithionite titration. It is important to note that the significant accumulation of FADH• during the course of the titration with NADH indicates that a substantial portion of the electron transfer from NADH-reduced EtfAB to bcd occurs in one-electron steps.

**Figure 3:**
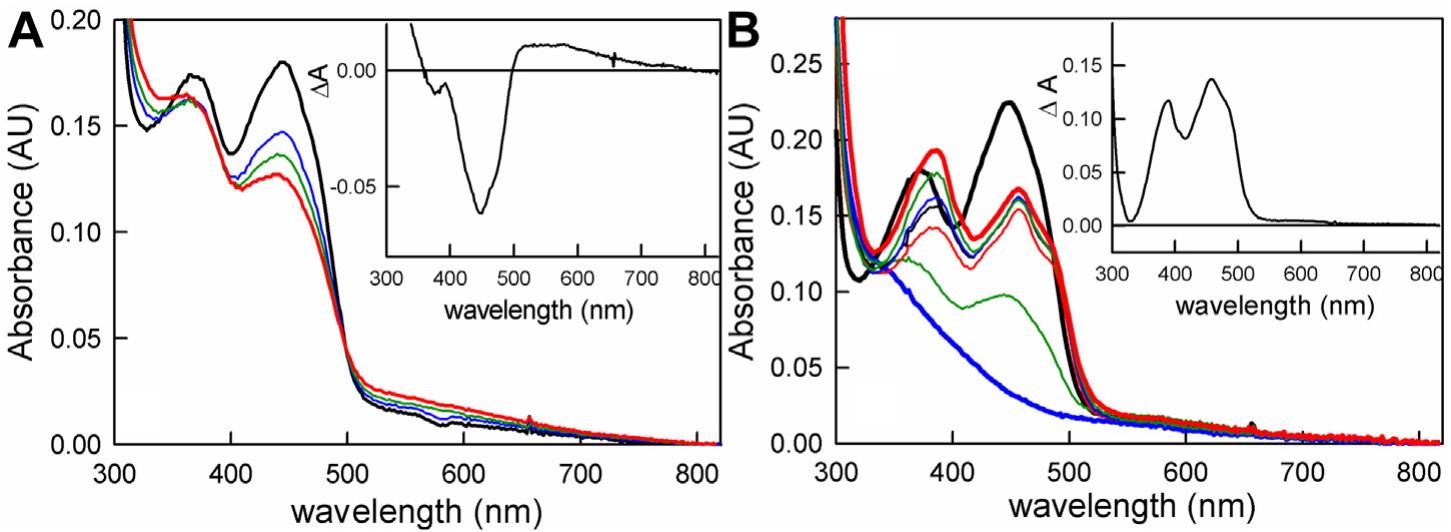
**Reductive titration of bcd with butyryl-CoA and oxidative titration with crotonyl-CoA.***A*, titration of 13 μM oxidized bcd (*black*) with butyryl-CoA. The spectrum in *red* reflects the greatest extent of bcd reduction with the addition of excess (100 μM) butyryl-CoA. *Inset*, the difference spectrum between the point of greatest enzyme reduction and that of oxidized enzyme. Negative features show flavin reduction with the maximum at 450 nm for FAD, and positive features above 500 nm show the formation of the bcd_ox_:butyryl-CoA charge-transfer complex with a maximum of 571 nm. *B*, reoxidation of 19 μM fully reduced bcd (*bold blue*) by crotonyl-CoA in 50 mM Tris-HCl, 150 mM NaCl, pH 7.5 to maximum reoxidation (*bold red*). *Bold black* shows fully oxidized bcd. *Inset*, difference spectrum, crotonyl-CoA oxidized minus reduced. Note the amount of the relative 377 nm peak is nearly the same as the 450 nm peak indicating the presence of FAD•^−^ and the small accumulation of long-wavelength absorbance is consistent with the small amount of bcd_red_:crotonyl-CoA charge-transfer complex. Experiments were performed in 50 mM Tris HCl, 150 mM NaCl, pH 7.5, 25 °C.

We also performed a reductive titration of bcd with butyryl-CoA (Fig. 3*A*). This is the reverse of the physiological direction but can be driven, if incompletely, by mass action in the presence of high concentrations of butyryl-CoA. Although the long-wavelength absorbance in the 500 to 600 nm range (Fig. 3, insets) closely resembles the accumulation of FADH•, EPR indicates negligible semiquinone accumulation (data not shown). We attribute the long-wavelength absorbance instead to a bcd_ox_•butyryl-CoA charge-transfer complex, by analogy to the well-characterized charge-transfer complexes of enzyme with crotonyl-CoA (20), acetoacetyl-CoA (15), and CoA persulfide (12) (see Fig. S1). A second charge-transfer complex involving reduced bcd and crotonyl-CoA is also formed transiently in the course of reacting reduced bcd with crotonyl-CoA, and the spectrum of this complex is very similar to that of the bcd_ox_•butyryl-CoA complex (Fig. S2).

Lastly, we titrated reduced bcd with crotonyl-CoA, *i.e.*, the reaction of physiological relevance to bifurcation as catalyzed by the intact EtfAB–bcd complex. As with the reduction of bcd by butyryl-CoA, the reaction does not progress to completion (Fig. 3*B*). In addition, there is a significant absorbance increase at 377 nm in comparison with the absorption decrease at 450 nm at the end of the reaction (compare Fig. 3*B* with Fig. 1*B*), consistent with the accumulation of the anionic semiquinone, FAD•^−^ rather than the neutral FADH• seen in the course of dithionite titrations. This suggests, as verified by EPR below, that substrate/product binding results in ionization of the semiquinone oxidation state.

### Kinetics of reduction and reoxidation of bcd

**Figure 4:**
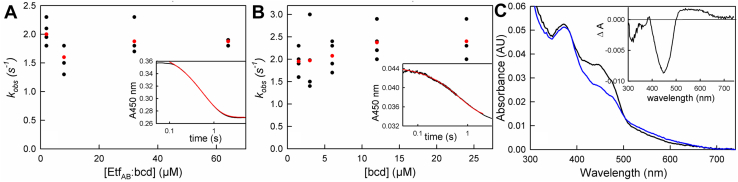
**The reaction of reduced EtfAB with oxidized bcd.***A*, reaction of prereduced EtfAB with oxidized bcd in a 1:1 ratio monitored at 450 nm. The average rates seen at each concentration are marked in red. The apparent *k*_*red*_ is 2 s^−1^ and is independent of absolute concentrations of the complex. *Inset*, a representative transient seen with 32 μM of EtfAB and bcd (*black*) and a single-exponential fit to the data with *k*_*obs*_ = 1.9 s^−1^ (*red*). *B*, the reaction of 3 μM prereduced EtfAB with increasing concentrations of oxidized bcd. The average rates of each concentration are marked in *red*, and again the concentration-independent *k*_*red*_ is 2 s^−1^. *Inset*, a representative transient at 3 μM Etf_AB_ and 3 μM (*black*) with a single-exponential fit with *k*_*obs*_ = 2.0 s^−1^. *C*, spectral changes associated with the reaction of 3 μM prereduced EtfAB with 3 μM bcd. The spectra seen immediately after mixing (*black*) and at the end of reaction (*blue*) are shown. *Inset*, the total spectral change associated with the reaction, with positive features above 500 nm reflecting the accumulation of bcd FADH•. Experiments were performed in 50 mM Tris-HCl, 150 mM NaCl, pH 7.5, 25 °C.

We next investigated the rapid-reaction kinetics of the reductive and oxidative half-reactions of bcd. This included the reaction of oxidized bcd by fully (*i.e.*, four-electron) reduced EtfAB, the reoxidation of reduced bcd by crotonyl-CoA, and the reduction of bcd by butyryl-CoA, all by stopped-flow spectrophotometry. Previous work had shown that reduction of EtfAB by NADH is fast with a hyperbolic dependence of *k*_*obs*_ on [NADH] and limiting *k*_*red*_ at high NADH of 590 s^−1^ (18). The reaction of oxidized bcd with prereduced EtfAB involves the transfer of one or two reducing equivalents from the et FAD of EtfAB to the bcd FAD. The distinct absorption spectra of the two flavins (given the red-shifted absorption of the et FAD) allows us to observe the reaction at 450 nm (18). The reaction of equimolar concentrations of fully reduced EtfAB and bcd (32 μM each) exhibits an apparent *k*_*red*_ of 2 s^−1^ at 25 °C. The process is a well-defined monophasic exponential, as is apparent from the quality of the exponential fit to the data shown in Figure 4*A*, inset. The observed rate constant is independent of the equimolar protein concentrations over the range 2 to 70 μM, indicating that the K_d_ for binding EtfAB to bcd is much lower than 2 μM. The observed rate constant also does not change when the reaction is performed under approximately pseudo-first-order conditions, reacting 3 μM prereduced EtfAB with up to 25 μM bcd, as seen in Figure 4*B*, suggesting that the observed kinetics do not arise from the second-order binding of the two proteins. Significantly, inspection of the observed spectral change at longer wavelengths indicates that there is substantial accumulation of FADH• in the course of the reaction (Fig. 4*C*). The magnitude of the absorbance increase indicates that some 40% of the total reduced bcd FAD is in the semiquinone form at the end of the reaction. This in turn means that a significant amount of the electron transfer from the reduced EtfAB to bcd occurs in a one-electron process. Forty percent undoubtedly underestimates the extent of one-electron transfer as some FADH• is very likely reduced subsequently on to the hydroquinone in a second one-electron process. Given that the reduction potentials of the Q/HQ of the et FAD in EtfAB are approximately 136 mV more negative than the SQ/HQ bcd FAD it stands to reason that the two-electron transfer would be thermodynamically quite favorable (17), but since both half-potentials are more negative than the crotonyl-CoA/butyryl-CoA couple, the existence of both one- and two-electron processes is not unexpected.

**Figure 5:**
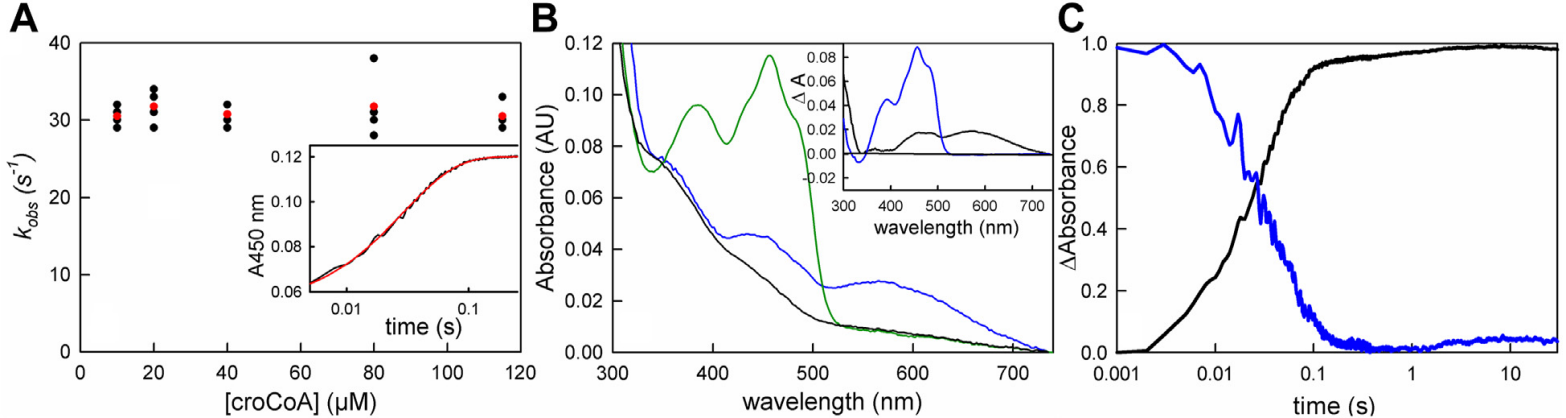
**The oxidation of bcd by crotonyl-CoA.***A*, reaction of 12 μM bcd with increasing [crotonyl-CoA] as monitored at 450 nm. The average rates of each concentration are marked in *red*. The apparent *k*_*red*_ is 30 s^−1^ and is independent of [crotonyl-CoA]. *Inset*, a representative transient seen with 112 μM crotonyl-CoA (*black*) and the single-exponential fit with *k*_*obs*_ = 30 s^−1^ (*red*). *B*, spectral changes associated with the reaction of 16 μM prereduced bcd and 112 μM crotonyl-CoA: *black*, fully reduced bcd; *blue*, the spectrum seen immediately after mixing (∼1 ms); *green*, the final spectrum at the end of the reaction. *Inset*, difference spectra showing the full formation of the bcd_red_:crotonyl-CoA charge-transfer complex (*black*) and full reoxidation of bcd (*blue*). *C*, the normalized kinetic transients seen at 450 nm, following flavin reoxidation (*black*) and at 571 nm following loss of the charge-transfer complex (*blue*) in the 112 μM crotonyl-CoA reacted with bcd. Reoxidation of bcd and the decay of the bcd_red_:crotonyl-CoA complex are seen to occur concomitantly. Reactions were performed in 50 mM Tris-HCl, 150 mM NaCl, pH 7.5, 10 °C.

The kinetics of the oxidative half of the reaction were next examined, reacting prereduced bcd with crotonyl-CoA, the experiment being performed at 10 °C given the fast kinetics that were observed. The observed kinetics were monophasic with a rate constant that was independent of [crotonyl-CoA] over the range 10 to 110 μM, with an apparent rate of 30 s^−1^ (Fig. 5*A*). The bcd_red_:crotonyl-CoA charge-transfer complex observed in the reductive titrations described above was fully formed in the mixing dead time of the stopped-flow apparatus, as shown in Figure 5*B* (blue), indicating extremely rapid and tight binding of crotonyl-CoA to bcd. The difference spectrum between the spectrum obtained immediately after mixing and that of the fully reduced enzyme is shown in the inset to Figure 5*B* to illustrate formation of the charge-transfer complex (black); the difference spectrum for the overall spectral change is also shown (blue). When reduced bcd is reacted with excess crotonyl-CoA, full reoxidation of the FAD is observed with loss of the charge-transfer complex occurring at a comparable rate, as shown in Figure 5*C*.

**Figure 6:**
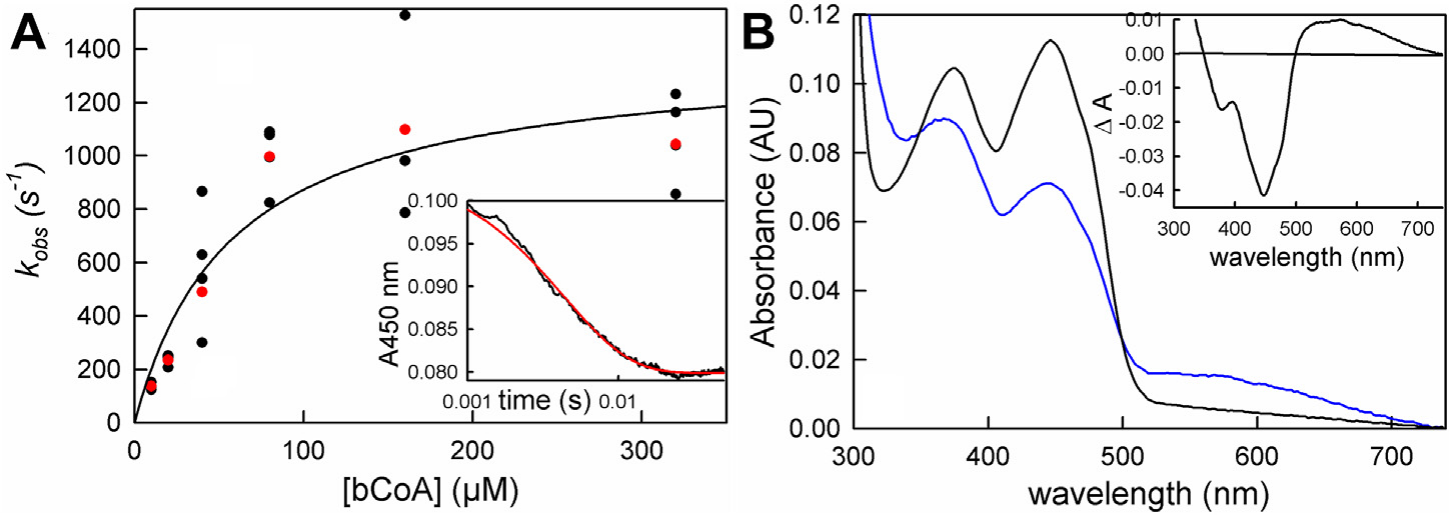
**The reduction of bcd by butyryl-CoA.***A*, reaction of 10 μM bcd with increasing [butyryl-CoA] monitored at 450 nm. The average rates of each concentration are marked in *red*. The K_d_ and limiting *k*_*red*_ from hyperbolic fits to the data are 60 μM and 1400 s^−1^, respectively. *Inset*, a representative transient of 80 μM butyryl-CoA out to 0.025 s. *B*, the reaction of 10 μM bcd with 640 μM butyryl-CoA: *black*, oxidized enzyme; *blue* is the spectrum at the end of reaction (∼2 ms). *Inset*, the difference spectrum associated with the overall reaction, where negative features represent flavin reduction, showing the full formation of the bcd_ox_:butyryl-CoA charge-transfer complex and reduction of FAD (*black*). Reactions were performed in 50 mM Tris-HCl, 150 mM NaCl, pH 7.5, 10 °C.

Finally, we examined the kinetics of bcd reduction by butyryl-CoA, working again at 10 °C given the fast rates that were observed. The transients were again monophasic, but unlike the reaction with crotonyl-CoA, the observed rate constant exhibited a hyperbolic dependence on [butyryl-CoA]. As shown in Figure 6*A*, hyperbolic fits of the plot of *k*_*obs*_ *versus* [butyryl-CoA] yielded an apparent K_d_ of 60 μM and limiting *k*_*red*_ of 1400 s^−1^. Given the unfavorable thermodynamics of the reaction noted above, the extent of reduction at lower concentrations of butyryl-CoA increased due to a mass action effect, but above 100 μM butyryl-CoA the extent of reduction became independent of butyryl-CoA. The reduction potential of the crotonyl-CoA/butyryl-CoA is −10 mV (17), and consistent with this we find that in order to poise the system for the butyryl-CoA/crotonyl-CoA equilibrium a ratio of 10:1 is required, as previously reported (14). Given this, the K_d_ determined from the hyperbolic plot must be considered an apparent value, but the limiting *k*_*red*_ is reliable. We note that, in this experiment, the fastest experimentally determined rate constant was 1000 s^−1^, at the limit of our stopped-flow instrument, and the value of 1400 s^−1^ was obtained by extrapolation of the hyperbolic plot. Consistent with the results of the equilibrium titration of oxidized bcd with butyryl-CoA (Fig. 3*A*), the reaction does not go to completion even at the highest concentrations of butyryl-CoA used, and a charge-transfer complex of butyryl-CoA with the substantial amount of enzyme that remains oxidized is seen at the end of reaction, as reflected in the extended absorption increase above 500 nm (Fig. 6*B*).

### Electron paramagnetic resonance characterization of flavin semiquinone

**Figure 7:**
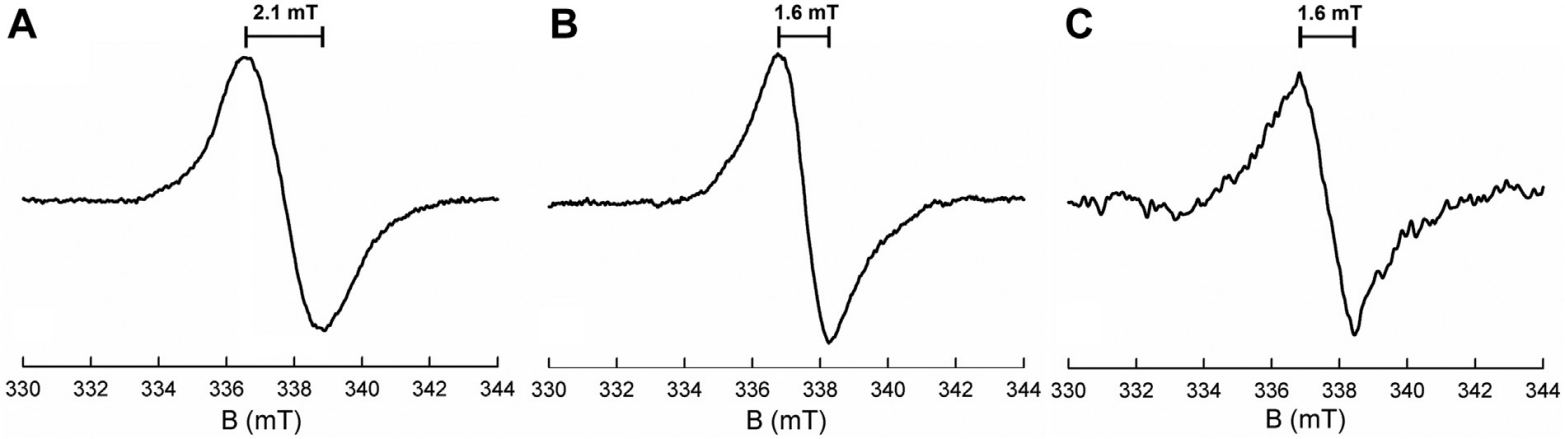
**X-band electron paramagnetic resonance spectrum of the semiquinone signal at 77 K.***A*, spectrum of 30 μM bcd titrated with sodium dithionite to maximum accumulation of FADH• as verified by maximum absorption at 571 nm at pH 6.0. The peak-to-trough linewidth is 21 G (2.1 mT), which correlates to the UV-visible as the neutral semiquinone. *B*, spectrum of 30 μM bcd fully reduced with sodium dithionite and reoxidized with 100 μM of crotonyl-CoA at pH 7.5. The linewidth is 16 G (1.6 mT) and correlates to the 377 nm absorbance seen in the reoxidation above (Fig. 4*B*). *C*, bcd, 100 μM, reacted with 1 mM butyryl-CoA at pH 7.5, as with the crotonyl-CoA reoxidized sample. The weak signal with linewidth measured at 16 G (1.6 mT) represents only a small amount of FAD•^−^ and correlates well with the absence of FADH• seen in the UV-visible spectra (Fig. 4*A*).

As indicated above, the et FAD of EtfAB exhibits a thermodynamically stable anionic semiquinone in the one-electron reduced state (18). On the other hand, bcd exhibits a neutral semiquinone in the course of dithionite titrations, particularly at lower pH values (Fig. 1). This is also the case for the bcd within the EtfAB–bcd complex (19, 21). FADH• and FAD•^−^ can be differentiated on the basis of their different EPR linewidths (22), which fall in the range 1.9 to 2.2 and 1.4 to 1.6 mT, respectively. When bcd is reduced by one equivalent in the absence of either crotonyl- or butyryl-CoA, a 2.1-mT linewidth is seen (Fig. 7*A*), which in conjunction with the observed long-wavelength absorbance (Fig. 1) unambiguously establishes the semiquinone to be the neutral FADH•. On the other hand, there is a large accumulation of absorbance at 377 nm when reduced bcd is reoxidized by crotonyl-CoA, suggesting formation of the anionic FAD•^−^ semiquinone (Fig. 3*B*). The resulting EPR signal, shown in Figure 7*B*, exhibits a linewidth of 1.6 mT, indicating that FAD•^−^ does indeed form in the presence of crotonyl-CoA. The intensity of the signal represents approximately 20% of the total enzyme as the FAD•^−^. The extent of formation of FAD•^−^ in the bound state also agrees well with the observed extent of reaction as shown in Figures 5 and 6. On the basis of these results, it is clear that binding of substrate/product results in ionization of the bcd semiquinone, a point discussed further below. In the case of the reductive half-reaction, the present results underscore the importance of one-electron processes in the course of electron transfer from EtfAB to bcd, as previously suggested in the context of structurally characterized motions of the et FAD-containing domain of EtfAB (6). The absence of flavin semiquinone accumulation in the course of reoxidation of reduced bcd by crotonyl-CoA is consistent with the reaction occurring cleanly in a two-electron process. It is important to note, however, that there is precedent for a one-electron process with an accompanying radical on the substrate in a similar system as shown in the reaction that has previously been characterized in the dehydration of 2-hydroxy-4methylpentanoyl-CoA by *C. difficile* (23).

Finally, in an attempt to establish whether the long-wavelength absorbance seen during the reduction of bcd by butyryl-CoA was due to the neutral semiquinone or a charge-transfer complex, we reduced 100 μM of bcd with 1 mM butyryl-CoA (Fig. 7*C*); there was no EPR evidence for FADH•, although a small amount of a signal with a linewidth of 16 G, representative of a very small percentage of FAD•^−^ (∼2%) formed during the course of reduction by butyryl-CoA, was detected (Fig. 7*C*). This indicates that the transient absorbance increases seen in Figures 5*B* and 6*B* are due to charge-transfer complexes and not neutral semiquinone.

## Discussion

In the present work, and consistent with earlier work (15), we see transient accumulation of neutral flavin semiquinone in the course of reductive titrations of bcd with sodium dithionite, as reflected in the accumulation of long-wavelength absorbance at intermediate levels of reduction; as expected, accumulation is greater at lower pH. Interestingly, some FADH• also accumulates in the course of reductive titrations with NADH in the presence of catalytic amounts of EtfAB, meaning that, although the EtfAB is expected to be fully reduced under the reaction conditions and therefore capable of direct two-electron reduction of bcd to the fully reduced hydroquinone there is considerable one-electron transfer to the bcd. On the basis of the magnitude of the transient absorption increase at long wavelength seen in the course of the reaction (Fig. 4*C*), the observed accumulation of FADH• is approximately 40% of the total enzyme flavin, and this undoubtedly underestimates the proportion of one-electron transfer occurring as some of the FADH• generated undoubtedly reduced on to the hydroquinone in a second one-electron event. In oxidative titrations with crotonyl-CoA and reductive titrations with butyryl-CoA, long-wavelength absorption again accumulates, although in both cases this is due to the formation of bcd_red_•crotonyl-CoA and bcd_ox_•butyryl-CoA complexes. Furthermore, while significant (20%) flavin semiquinone accumulates, it is the anionic FAD•^−^ rather than the neutral FADH• (see Fig. 7*B*), as confirmed by EPR. Similarly, long-wavelength absorption accumulates in the course of titrations of reduced bcd with crotonyl-CoA, again due to formation of charge-transfer complexes, and again the semiquinone that accumulates is FAD•^−^ rather than FADH•.

The observation of neutral FADH• in the absence of substrate/product and the anionic FAD•^−^ in their presence indicates that their binding results in ionization of the semiquinone. Similar behavior has been observed with, *e.g.*, trimethylamine dehydrogenase, where the anionic semiquinone is observed when the enzyme is radiolytically reduced while the neutral form is formed after the enzyme is inactivated by ferricenium and subsequently reduced (24). In the case of bcd it has been reported that the binding of the substrate analogue, acetoacetyl-CoA, results in a shift in the reduction potential of the enzyme by −100 mV of the free enzyme (15), illustrating the effect of substrate binding on the environment of the flavin.

That the absence of anionic semiquinone accumulation in the stopped-flow experiment seen here (Fig. 5*B*) contrasts with its significant accumulation in the course of the titration of reduced bcd with crotonyl-CoA (Fig. 3*B*) deserves comment, particularly given that the final concentrations of crotonyl-CoA in the two experiments are the same (100 μM). Having confirmed that the observations in both the titration and stopped-flow experiments are fully reproducible, we have performed two additional experiments. In the first, equal volumes of solutions of reduced bcd and crotonyl-CoA were mixed into an anaerobic cuvette, mimicking after a fashion the conditions of the stopped-flow experiment. No semiquinone, anionic or neutral, was observed, and as in the stopped-flow experiment the reduced bcd was fully oxidized in a two-electron process (Fig. S3). That the long-wavelength absorbance seen transiently in the course of reoxidation was due to the bcd_red_•crotonyl-CoA charge-transfer complex and not the neutral semiquinone was confirmed by EPR (data not shown). In the second experiment, reduced bcd was mixed with varying small volumes of concentrated crotonyl-CoA in the stopped-flow apparatus, in a manner that mimicked additions in the course of the oxidative titration. As in the case of the titration experiment, a significant amount of anionic semiquinone was indeed observed, as reflected in the transient increase in absorbance at ∼380 nm and confirmed by EPR (Fig. S4). We conclude that reoxidation of reduced bcd by crotonyl-CoA is normally a strict two-electron process, as expected and as seen in the stopped-flow experiment, but that due to a mixing artifact when mixing bcd_red_ with small volumes of concentrated crotonyl-CoA some semiquinone does accumulate in the course of oxidative titrations. The above notwithstanding, it is unequivocal that, under a variety of conditions when semiquinone does accumulate in the presence of crotonyl-CoA, it is the anionic rather than neutral semiquinone that is observed. We note that the tendency of crotonyl-CoA binding to facilitate ionization of the flavin N5-H would facilitate oxidation of both FADH^−^ and FAD•^−^ by crotonyl-CoA.

Electron transfer from prereduced EtfAB to oxidized bcd is slow, with an observed *k*_*red*_ of 2 s^−1^ that is independent of the concentration of bcd above 2 μM (Fig. 4). That the rate constant is independent of [bcd] indicates that the EtfAB–bcd complex forms with a K_d_ smaller than 2 μM and forms within the mixing dead time of the stopped-flow apparatus. The observed exponential behavior of the absorbance change thus reflects the rate of *intramolecular* electron transfer. As indicated in the Introduction, the domain containing the electron-transferring FAD of EtfAB lies with its flavin close to the bifurcating FAD in the absence of bcd, while in the intact complex the domain containing the electron-transferring FAD has swung around so that its flavin now lies in proximity to the flavin of bcd. It has been suggested that this motion serves to gate electron transfer in the course of catalysis (6), and it may well limit the rate of electron transfer to bcd. We note that, although a rate of 2 s^−1^ is significantly less than that of the reduction of EtfAB *via* NADH (*k*_*red*_ = 590 s^−1^ (18)), it is still approximately an order of magnitude faster than the reported *k*_*cat*_ for ferredoxin reduction, which is 0.29 s^−1^ (5). The spectral change associated with electron transfer bears the signature long-wavelength absorbance of FADH• (Fig. 4*C*), indicating again a substantial amount of bcd reduction is a one-electron event. Sequential one-electron transfer events to bcd would serve to gate electron transfer so as to prevent low-potential reducing equivalents from progressing down the high-potential pathway (a strongly thermodynamically favorable process, but one that would short circuit bifurcation). As has been hypothesized (6), the enzyme complex during turnover is in a partially reduced state *in vivo* and the ability to clear electrons from the high-potential pathway and maintain a poised, partially reduced state, would aid in ensuring the fidelity of bifurcation. Likewise, if the EtfAB–bcd complex was to be completely and quickly reoxidized then the reducing equivalents generated from the reduction by NADH would proceed down the much more favorable high-potential pathway and again result in stopping bifurcation. This ensures that the enzyme complex can continue to facilitate turnover whenever ferredoxin is present and continue to engage in the reverse oxidation pathway. Although electrons can be transferred in pairs between FADs, this is not obligatory, as exemplified in simple ETFs that function physiologically in one-electron transfers between their physiological partners. In this way, the enzyme can also have a way to protect itself from newly generated reducing equivalents that could easily rereduce the newly oxidized bcd.

The oxidation of reduced bcd by crotonyl-CoA occurs with an observed rate constant of 30 s^−1^ and is independent of [crotonyl-CoA] above 10 μM. The reaction is monophasic at 450 nm, but at 600 nm there is clear evidence of the formation and decay of bcd_red_:crotonyl-CoA and bcd_ox_:butyryl-CoA charge-transfer complexes. While previously observed (12, 20), the present work establishes that these complexes are kinetically competent to be catalytic intermediates. The reverse reaction, the reduction of bcd by butyryl-CoA, is thermodynamically unfavorable but can be forced by mass action at high concentrations of butyryl-CoA, although reduction does not go to completion. The kinetics exhibit hyperbolic dependence on [butyryl-CoA], with an observed *k*_*red*_ of ∼1400 s^−1^. Again, the transients seen at 450 nm are monophasic but those at longer wavelengths indicate the formation of the same charge-transfer complexes as seen in the reverse (*i.e.*, physiological) direction.

**Figure 8:**
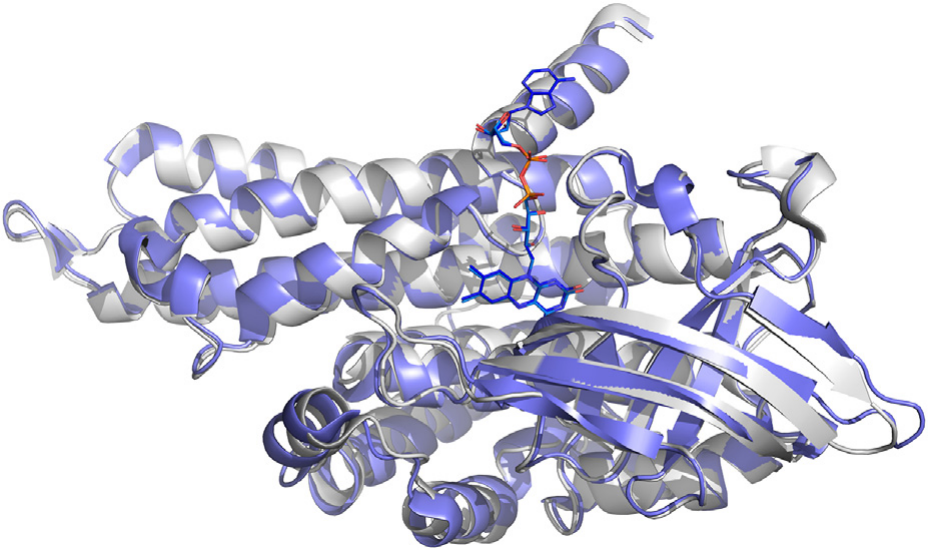
**Alignment of the structures of EtfAB-bound bcd (Protein Data Bank****5Ol2****) and free bcd (Protein Data Bank****1BUC****).***Blue* is the structure of the uncomplexed bcd from *M. elsdenii* aligned with the bcd component from the *C. difficile* structure.

We note that the structure of the bcd component in the *C. difficile* structure and the structure of the free bcd from the crystal structure of *M. elsdenii* are virtually identical, as illustrated in Figure 8, with an rms deviation of C_α_ between the two structures of 0.585 Å. Given the lack of conformational changes in bcd on complex formation with EtfAB, we conclude that the observed kinetics of isolated bcd with crotonyl-CoA and butyryl-CoA are relevant to the complex as well.

## Experimental procedures

### Organisms and growth conditions

Plasmids derived from pASG-IBA3 (IBA GmbH) containing the *M. elsdenii* bcd (*Mels_2128*) and EtfAB (*Mels*_2126 + *Mels*_*2127*) and a C-terminal Strep II tag were constructed as described by Chowdhury *et al.*, 2015. Plasmids were transformed into *Escherichia coli* BL21 cells with growth conditions as reported (17). Cells from glycerol stocks were placed in 10 ml Terrific Broth (TB) with 100 μg/ml of ampicillin and grown overnight at 37 °C and shaking at 220 rpm. Large-scale growth was performed in 2 l of TB in a 6-l Erlenmeyer flask with 100 μg/ml of ampicillin. The flasks were inoculated with the preculture and grown for ∼6 h at 37 °C and shaking at 180 rpm. When the *A*_600 nm_ reached 0.5, the temperature was reduced to 21 °C and shaking continued until absorbance reached 0.8 at which time the cells were induced with 0.1 mM anhydrotetracycline and grown overnight. Cells were harvested by centrifugation for 20 min at 5500*g* at 4 °C. The resulting pellets were then flash frozen in liquid nitrogen and stored at −80 °C until used for purification.

### Purification of *M. elsdenii* EtfAB and bcd

Purification of EtfAB and bcd was performed at 0 to 4 °C under aerobic conditions. A procedure described (17) was modified to include the addition of excess FAD during the washing steps. Cells were resuspended in 50 mM Tris-HCl, 150 mM NaCl, pH 7.5 (buffer A) with 1 mM NaF, 1 mM benzamidine, 0.5 mM phenylmethylsulfonyl fluoride (PMSF), and catalytic amounts of DNase I and lysozyme, and then incubated for 60 min before breaking. Cell lysis was performed *via* 1 to 2 passages through a French Pressure Cell at 10,000 psi. To remove cell debris, the lysate was centrifuged for 60 min at 200,000*g*. The supernatant was then flash frozen in liquid nitrogen and stored at −80 °C. Thawed supernatant was bound batchwise with 6 ml of Strep-Tactin XT Superflow resin (IBA GmbH) pre-equilibrated with 50 mM Tris-HCl, 150 mM NaCl, pH 7.5. The column was washed with 30 column volumes of buffer A with 100 μM FAD. After washing, elution was accomplished using 50 mM biotin in 50 mM Hepes (pH 7.5) at 0.5 ml/min. Protein was then concentrated to a final volume of less than 1 ml *via* Amicon Ultra 4 (Millipore). After concentration, bcd was made anaerobic as described (17) *via* five vacuum cycles on an Argon train. Sodium dithionite was added in excess in order to fully reduce the protein and remove protein-bound persulfide CoA (12). The reduced protein was then centrifuged at 10,000*g* for 30 min; excess FAD/dithionite was removed and the protein buffer exchanged into buffer A using an 8.3-ml PD-10 (GE Healthcare) column equilibrated in said buffer A. Purified protein was then aliquoted, frozen, and stored at −80 °C for future use.

### UV-visible absorbance measurements

A Hewlett-Packard 8452A diode-array spectrophotometer equipped with a thermostatted cell holder was used for all static anaerobic titrations as well as routine absorbance measurements. Concentrations for EtfAB and bcd were calculated using molar extinction coefficients of ε_450_ = 21.1 mM^−1^ cm^−1^ and ε_450_ = 11.8 mM^−1^ cm^−1^, respectively, as reported (8). Static titrations were performed in a quartz anaerobic sidearm cuvette sealed with a rubber septum. Protein samples with glucose oxidase from *Aspergillus niger* (Sigma Type VII, typically 80 nM) and bovine liver catalase (Sigma C-40, typically 8 nM) were made anaerobic *via* the Argon dry train on ice. After each sample was made anaerobic, glucose was added from the sidearm to a final concentration of 2 to 10 mM, which would scrub the solution of any residual O_2_ as described (17). The reductants or oxidants, in this case NADH (Thermo Scientific J61638.03), sodium dithionite (Reagents C2316300), butyryl-CoA (Sigma B1508), or crotonyl-CoA (Sigma C6146), were also made anaerobic on the Argon train. The reductants or oxidants were then added to the sealed sidearm cuvette by way of a Hamilton syringe piercing the rubber septum. The interface between the needle and the septum was sealed using Apiezon N vacuum grease. During collection of titration data, an oxidized spectrum was taken; then after each addition of reductant aliquot, two spectra would be taken, one directly after addition and one 5 to 10 min following addition to confirm complete reaction. Aliquots of reductant were added until the measured spectra matched the published fully reduced enzyme spectrum.

### EPR spectroscopy

EPR spectra were recorded with a Bruker Magnettech ESR500 spectrometer running acquisition software, ESRStudio 1.80.0, and using a 50 mL liquid nitrogen Dewar flask to maintain a temperature of 77 K. Parameters were as follows: modulation amplitude of 0.6 mT and power of 0.02 mW. To prepare samples, protein was made anaerobic at room temperature on an anaerobic train and transferred into a sealed EPR tube previously flushed with argon. Anaerobic sodium dithionite, crotonyl-CoA, or butyryl-CoA was then added to the sealed EPR tube *via* a Hamilton syringe. To verify the presence of semiquinone, the samples were checked by UV-visible spectroscopy and then immediately frozen in a bath of ethanol and dry ice. Samples were then stored in liquid nitrogen to await EPR spectroscopy. Samples reduced with sodium dithionite were made in 50 mM MES, 150 mM NaCl, pH 6.0 in order to ensure the maximum signal accumulation and yielded the neutral semiquinone FADH•. Samples made in the presence of crotonyl-CoA and butyryl-CoA were prepared in 50 mM Tris-HCl, 150 mM NaCl, pH 7.5, then reacted with prereduced and oxidized bcd, respectively. This yielded the anionic semiquinone FAD•^−^. Total amount of semiquinone accumulation was compared with *A. vinelandii* flavodoxin standard and quantified as described (16).

### Rapid-reaction kinetics

Measurements of rapid-reaction kinetics were observed using an Applied Photophysics SX-20 stopped-flow spectrophotometer equipped with either a photodiode array (PDA) or photomultiplier tube (PMT) detection with the acquisition software ProData SX 2.2.5.6. Samples containing bcd and EtfAB were made anaerobic in buffer A with catalytic amounts of glucose oxidase and catalase. Anaerobicity was achieved in an iced tonometer on an anaerobic train before mounting onto the stopped-flow instrument. Using the diode array detector, spectra in the range 230 to 740 nm were obtained. Kinetic transients at a given wavelength, typically at 450 nm, were extracted from the PDA data set and used for rate determination. With the photomultiplier detector, kinetic transients at specific wavelengths were obtained for the fastest of the reactions. All data were collected at 25 °C except for the reoxidation *via* crotonyl-CoA and the reduction *via* butyryl-CoA, which were collected at 10 °C. For analysis of the fast phases of FAD reduction, only the first 10 to 500 ms immediately following mixing were used. Time courses were fit to the sum of two exponentials by a nonlinear least squares regression analysis using the equation:

$$A_{t}=A_{\infty }\pm \sum A_{n}\;\exp\left(-t/k_{n}\right)$$

where n is the number of kinetic phases. ProData Viewer 4.2.0 software was used to analyze the time courses. NADH concentration was plotted against *k*_*obs*_, the observed rate constant, and the data were fitted to the following equation:

$$k_{obs}=k_{red}\left[S\right]/\left(K_{d}+\left[S\right]\right)$$

The resultant fit allowed for the determination of the limiting rate constant for reduction, *k*_*red*_, as well as the apparent dissociation constant, K_d_. The reaction of the prereduced EtfAB and oxidized bcd was performed and analyzed as described above. EtfAB was prereduced with sodium dithionite and reacted with oxidized bcd in two schemes at 25 °C: first at a 1:1 ratio of both proteins in the concentration range of 2 to 70 μM and second with 3 μM of prereduced EtfAB with increasing concentrations of oxidized bcd in the range of 3 to 24 μM. The reduction of bcd was fit to a single exponential.

The reaction of oxidized bcd with butyryl-CoA and that of reduced bcd with crotonyl-CoA were each monitored at 10 °C given the faster kinetics of the former. The photomultiplier detector was used to follow reduction of bcd with increasing concentrations of butyryl-CoA in order to more accurately observe the fastest portion of the reaction. With the photodiode array detector, the majority of the reaction with excess butyryl-CoA was complete in the mixing dead time of the stopped-flow instrument (*ca.* 1 ms) and the presence of the charge-transfer complex between oxidized bcd and butyryl-CoA was observed at longer wavelengths (maximum at 571 nm). The full wavelength spectral changes of the reaction of reduced bcd (prereduced with sodium dithionite) and crotonyl-CoA were monitored with the diode array detector. To determine the endpoint of the reaction, kinetic traces extracted from the PDA dataset at 450 and 571 nm were used to monitor the reoxidation of FAD and the decay of the charge-transfer complex, respectively. Both traces were well described by single exponential fits.

## Data availability

Data not contained in the article (protein gels, absorption spectra, and raw titration data) are available on request to the corresponding author (russ.hille@ucr.edu).

## Supporting information

This article contains supporting information.

## Conflicts of interest

The authors declare that they have no conflicts of interest with the contents of this article.
