# Supplementary material for: Rapid-reaction kinetics of the butyryl-CoA dehydrogenase component of the electron-bifurcating crotonyl-CoA-dependent NADH:ferredoxin oxidoreductase from Megasphaera elsdenii
Source: J Biol Chem. 2023 May 21;299(7):104853. doi: 10.1016/j.jbc.2023.104853 (PMC10320503; doi:10.1016/j.jbc.2023.104853)
Supplement: Figs. S1–S4 [file mmc1.docx]

**Rapid-reaction kinetics of the butyryl-CoA dehydrogenase component of the electron-bifurcating crotonyl-CoA-dependent NADH:ferredoxin oxidoreductase from *Megasphaera elsdenii*.**

Wayne Vigil Jr. ^1^, Derek Nguyen, Dimitri Niks and Russ Hille

Department of Biochemistry, University of California, Riverside, Riverside CA 92521

Supplemental Figures


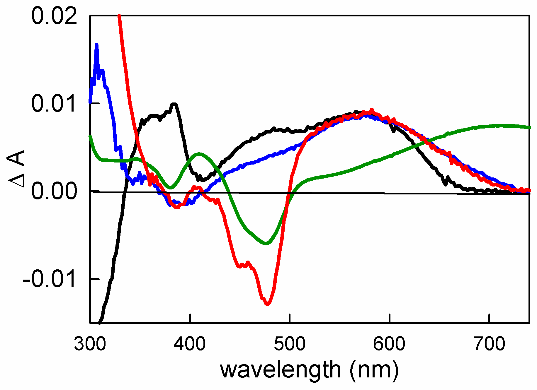


Figure 1 Long wavelength absorbance of bcd. Black is the FADH• as extracted from the sodium dithionite titration at pH 6.0. The main absorbance maxima are 370 nm, 470 nm and at 574 nm. All subsequent pure spectra were obtained by selecting points via inspection as done in Vigil et al 2021 during the course of titration or reaction. Blue is the bcd_red_:crotonyl-CoA CTC which was extracted from the rapid reaction kinetics experiment in Figure 6. The absorption maxima centered at 574 nm is in the same as the FADH• signal but is missing the 370 nm maxima. Green is the persulfide-CoA bound bcd as seen during purification, the absorbance maxima is centered around 700 nm. Lastly, red is the difference between oxidized bcd and then mixed with excess acetoacetyl-CoA, again we find an absorbance maximum at 574 nm, the negative features at 470 nm and 450 are a result of a shift in the FAD absorbance upon binding.


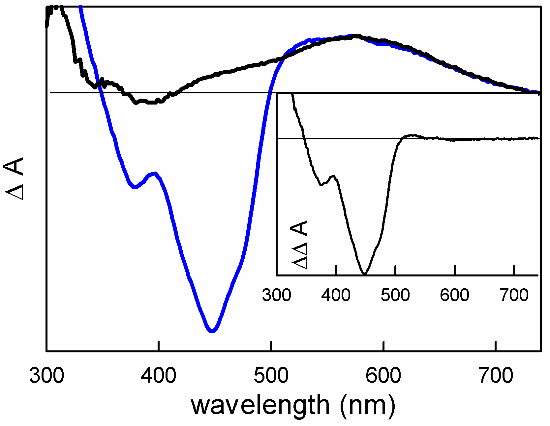


Figure 2 CTC spectra of bcd and the appropriate crotonyl/butyryl-CoA. Black spectra resulting from the binding of reduced bcd and crotonyl-CoA after 1 ms showing full formation of the bcd_red_:crotonyl-CoA CTC upon binding. Blue shows the absorbance from reacting oxidized bcd and butyryl-CoA, bcd_ox_:butyryl-CoA CTC, negative features are a result of FAD reduction in bcd during the mixing dead time of the stopped flow. Inset, is the difference spectra of bcd_red_:crotonyl-CoA CTC minus bcd_ox_:butyryl-CoA CTC, the unperturbed FAD spectra seen in the resulting difference spectra is illustrates that the two CTCs are indistinguishable from each other at any reasonable concentration.


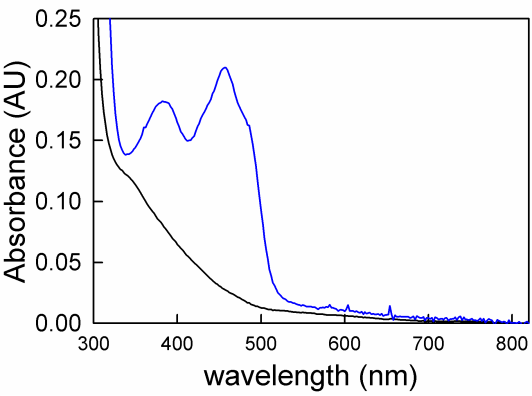


**Figure 3 Mimicking reoxidation of bcd by crotonyl-CoA with stopped-flow mixing in an anaerobic cuvette.** In a Hewlett-Packard 8452A diode-array spectrophotometer, a 500 µL solution of 10 µM bcd was titrated to max reduction by sodium dithionite in an anaerobic cuvette, then is taken up into a 500 µL Hamilton syringe. Another Hamilton syringe filled with 500 µL of 200 µM crotonyl-CoA pierces the anaerobic cuvette, both syringes are simultaneously ejected into the anaerobic cuvette and spectra are taken in kinetics mode, mimicking the equal volume mixing seen in the stopped-flow. The black spectrum shows the fully reduced bcd and the blue spectrum shows reoxidized bcd after mixing with crotonyl-CoA, without the presence of FAD•^-^.


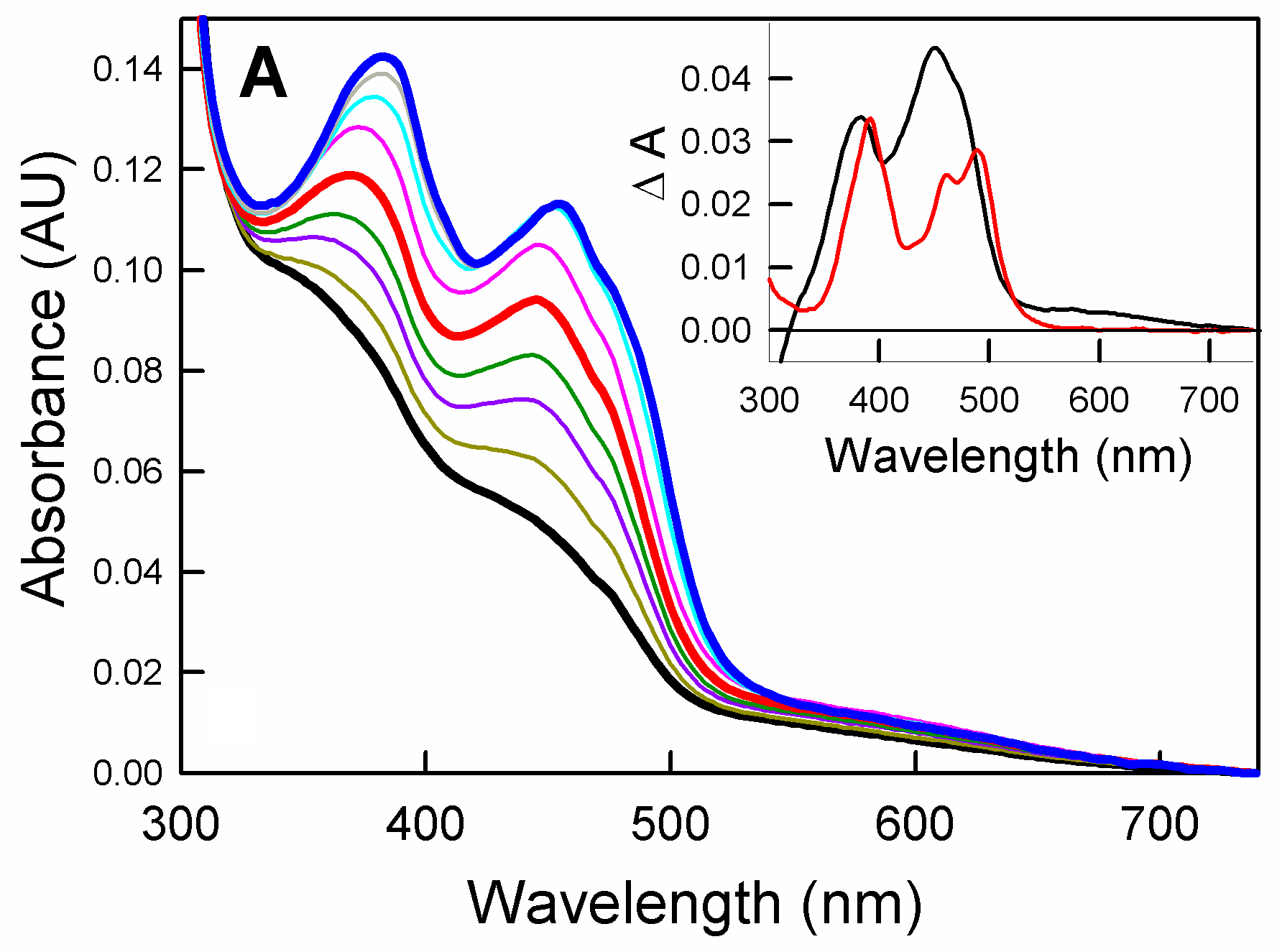

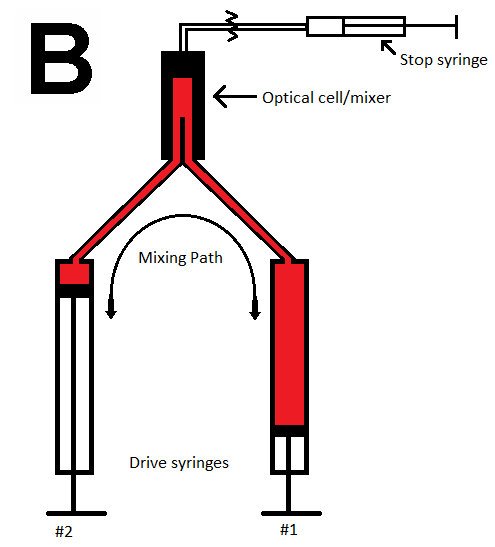


**Figure 4 Mimicking an oxidative titration of bcd with crotonyl-CoA in the stopped-flow apparatus.** A. Oxidative titration of 16 µM reduced bcd (bold black) by crotonyl-CoA in 50 mM Tris 150mM NaCl pH 7.5 to maximum reoxidation (bold blue). Inset, difference spectra of the bold red minus reduced spectra, showing flavin reoxidation (black), and the difference spectra of the bold blue minus bold red spectra, showing formation of FAD•^-^ (red). B. Diagram of the stopped flow apparatus. Red shows the mixing path between the two drive syringes, passing through the optical cell/mixer. The optical cell/mixer is blocked off to the stop syringe via a 2-way valve. The sample loading syringes are directly connected to their respective driving syringes, but this connection is blocked off during mixing via another 2-way valve.

Methods

Absorbance measurements were observed using the same PDA stopped-flow spectrophotometer and anaerobicity was achieved as mentioned in the methods of the rapid reaction kinetics. The difference in the use of the stopped-flow apparatus in the titration mimic experiment is in the mixing of the protein and titrant, as well as the method of pushing the sample into the optical cell. 2 mL of protein (bcd) solution is loaded into drive syringes #1, and titrant (crotonyl-CoA) is kept in the sample loading syringe #2. First, bcd is manually forwarded (without gas pressure) from drive syringe #1 into the optical cell to obtain the starting spectrum of the reduced protein. Next, a small amount of crotonyl-CoA stock is transferred from the sample syringe #2 into the drive syringe #2 (~20 µL). As described in the diagram, the solution is mixed between the two connected drive syringes through the optical cell multiple times, effectively mimicking a single titration addition. A static spectra was collected to observe the reoxidation of protein after mixing and after a few minutes to see the equilibrated spectra. This is repeated until the bcd is completely oxidized by crotonyl-coA. To correct any differences of dilution, the Supplemental Figure 5A spectra are scaled to match the original concentration.
